# Clinico-histopathological review of 255 patients who underwent parotidectomy for pleomorphic adenoma: a 10-year retrospective study—a proposal for an optimal diagnostic and therapeutic algorithm for patients with recurrent pleomorphic adenoma

**DOI:** 10.1007/s00405-023-07897-y

**Published:** 2023-03-06

**Authors:** Federica Zoccali, Fabrizio Cialente, Andrea Colizza, Massimo Ralli, Antonio Greco, Marco de Vincentiis

**Affiliations:** 1 Department of Sense Organs, Sapienza University of Rome, Viale del Policlinico 155, 00186 Rome, Italy; 2 Department of Oral and Maxillofacial Sciences, Sapienza University of Rome, Rome, Italy

**Keywords:** Benign tumor, Facial nerve, Parotidectomy, Recurrent pleomorphic adenoma, Salivary gland

## Abstract

**Purpose:**

Pleomorphic adenoma (mixed tumor) is the most common neoplasm of the parotid gland and one of the most frequent types of salivary gland tumor, generally with benign behavior and relatively slow growing. The adenomas could arise from the superficial, deep or from both superficial and deep parotid’s lobes.

**Methods:**

The aim of this review is to retrospectively analyze the surgical management of patients with pleomorphic adenoma of the parotid gland performed at the Department of Otorhinolaryngology (Department of Sense Organs of “Azienda Policlinico Umberto I” in Rome), from 2010 to 2020, with a focus on the percentage of recurrence and on the complication related to surgery to suggest an optimal diagnostic and therapeutic algorithm for patients with recurrent pleomorphic adenoma. The analysis of the complications observed in case of different surgical approaches was performed using the *X*^2^ test.

**Results:**

The choice of a surgical approach (superficial parotidectomy—SP, total parotidectomy—TP, extracapsular dissection—ECD) depends on several elements, such as the location and the size of the adenoma, the availability of existing technical facilities and the professional experience of the surgeon. A transient facial palsy was present in 37.6%, 2.7% reported a permanent facial nerve palsy, 1.6% developed a salivary fistula, 1.6% a post-operative bleeding and 2.3% showed Frey Syndrome.

**Conclusion:**

The surgical management of this benign lesion is required, even in asymptomatic cases, to prevent the progressive growing and to reduce the risk of malignant transformation. The goal of surgical excision is to obtain the complete resection to minimize the risk of tumor recurrence and avoiding facial nerve disability. Therefore, an accurate preoperative study of the lesion and the choice of the most appropriate surgical treatment are essential to minimize the rate of recurrence.

## Introduction

Parotid gland tumors account for almost 80% of all salivary gland tumors (SGT) [1]. Pleomorphic adenoma (PA) is the most common benign SGT, concerning both adults and children, followed by Warthin tumor (WT), also known as papillary cystadenoma lymphomatosum. To date, PA represents 85% of all salivary gland neoplasms, 60% of the benign tumors of the parotid gland and 3% of all head and neck tumors [2, 3]. This tumor usually arises between the fourth and the fifth decade, with a female predilection. No racial prevalence is reported. Although it usually presents itself as unilateral, it can also appear bilaterally. According to the most relevant literature, the majority of PA is found in the superficial lobe of the parotid gland (90% of lesions), while the deeper lobe is involved in less than 10% of cases [4]. PA, albeit benign, can relapse with recurrence rates of up to 6.8% [5]. Tumor factors that may have an influence on recurrence include its size, the histopathological subtype, the presence of satellite nodules and the inadequacy of removal. The literature shows that recurrences are often followed by an increased possibility of further ones and by the risk of a malignant transformation reported as 3.3% [3].

The diagnostic workup and treatment planning involves ultrasonography (US), magnetic resonance imaging (MRI), computed tomography (CT) and fine-needle aspiration cytology (FNAC). Recently, the introduction of contrast-enhanced ultrasound (CEUS) and elastography have contributed to improve the preoperative diagnosis of SGT [6]. Incisional biopsies are contraindicated due to the risk of tumor spreading, which could produce the recurrence of pleomorphic adenoma or malignancies [2]. Nevertheless, post-operative histology is still considered the gold standard for identifying the malignancy or the benignity of a lesion.

While the commonly preferred treatment requires the surgical excision of the neoplasm, the type of technique to be used is still debated. Before the 1940s, the standard surgical choice consisted of the enucleation of the tumor, with a significant recurrence rate (from 20 to 45%). From the 1950s, the partial or complete superficial parotidectomy represents the most used surgical technique [7].

The isolation of the facial nerve from the parenchyma constitutes the most difficult aspect of a parotidectomy and complications are mostly caused by this procedure. Nevertheless, intentional partial facial nerve resection may be performed depending on the histological type or on the extension of the tumor [8].

The aim of this retrospective study is to describe the experience of our University Hospital on the management of PA, comparing extracapsular dissection and superficial/complete parotidectomy, investigating the complication rates and evaluating the percentage of recurrence over a 10-year period.

## Materials and methods

In this study, we evaluated retrospectively 255 patients who underwent to parotid gland surgery between 2010 and 2020 at the Department of Otorhinolaryngology “Azienda Policlinico Umberto I” of Rome. The diagnosis was assessed by ultrasonography (US and CEUS), head and neck computed tomography (CT) or magnetic resonance (MRI) and fine-needle aspiration cytology (FNAC).

The inclusion criteria were:

1. Availability of information about demographic features of patients.
2. Information about preoperative exams (US, CT, MRI and FNAC).
3. Information on surgical technique for each patient.
4. Post-operative histopathological findings confirm the diagnosis of PA.
5. Information about follow-up.

The exclusion criteria were:

1. Other histological diagnosis (WT and malignant cancers).
2. Patients who underwent previous surgery on parotid gland.
3. Preoperative impairment of facial nerve.
4. Post-operative histopathological findings different from PA.
5. Patients lost during follow-up.

Considering these criteria, 255 patients were included among more than 480 parotidectomies performed from 2010 to 2020.

For each patient, we recorded demographics characteristics such as gender, age, past medical history, drinking and smoking habits and ethnicity, tumor location (superficial, deep or both parotid lobes), presence of complications due to the tumor invasion of the adjacent structures (frequently a sign of malignancy) and size.

In this work, we enrolled patient who underwent three main surgical technique: extracapsular dissection (ECD), superficial parotidectomy (SP), or total parotidectomy (TP).

The choice of surgical technique—whether extracapsular dissection (ECD), partial superficial parotidectomy (PSP), complete superficial parotidectomy (CSP), partial superficial/deep lobe parotidectomy (PSDP) or total parotidectomy (TP)—depended on tumors’ size and extension and to surgeons’ experience. A superficial parotidectomy approach was the most frequently applied and the Nerve Intraoperative Monitoring (NIM) of the facial nerve was used in each patient since 2013.

All post-operative complications (such as facial nerve injury, Frey Syndrome, post-operative bleeding, and salivary fistula) were recorded. Informed consent was acquired from all patients before surgical procedures and the study was approved by our Institutional Review Board.

Our study was performed in accordance with the Declaration of Helsinki and all the collected data were anonymized.

The statistical difference of the recurrent rates and complications between ECD and SP was performed using the *X*^2^ test. The chosen level of statistical significance was *P* < 0.05.

## Results

We enrolled a total of 255 cases of confirmed PA during the study period: 127 patients were males and 128 were females. The mean age at diagnosis was 56.92 years (range 21–84 years). All patients were of Caucasian race. A history of smoking was present for 55% of the patient population. PA was localized in the superficial lobe in 54.2% of cases and in the deep lobe in 8.4%, while it involved both lobes in 37.4% of cases.

The most common sign of onset was represented by a swelling in the parotid gland region (87%), followed by pain with a preauricular mass (4.9%) and pain without preauricular mass (2.4%). A parotid gland mass was accidentally discovered as incidental findings during imaging in 5.7% cases. No lymph nodes involvement was diagnosed in any of the patients. In Table 1, the main demographical and parotid gland disease characteristics of enrolled patients are resumed.

**Table 1:** Demographics and clinical features

Characteristic	Number (% or range)
Mean age at surgery	56.9 (21–84)
Sex	
Male	127 (49.8%)
Female	128 (50.2%)
Location of tumor	
Superficial lobe	54.2%
Deep lobe	8.4%
Both lobes	37.4%
Sign of onset	
Swelling	87%
Pain	4.9%
Pain + swelling	2.4%
No signs	5.7%

The 85% of patient have US like preoperative exam and FNAC was performed in 88.7% of patients. In our study, FNAC performed using ultrasound guidance had an overall sensitivity of 72% and a specificity of 99%, according to current literature [9, 10]. The findings were benign in 93% of these patients and malignant in 5%; the remaining 2% of samples were inconclusive. In the 2% of patients with suspect of malignancy, we also performed a CT scan with contrast agent and 3% had a preoperative MRI with gadolinium-based contrast agent. By these exams, we excluded preoperatively the suspect of malignancy or inconclusively FNAC.

### Surgical approaches

**Fig. 1:**
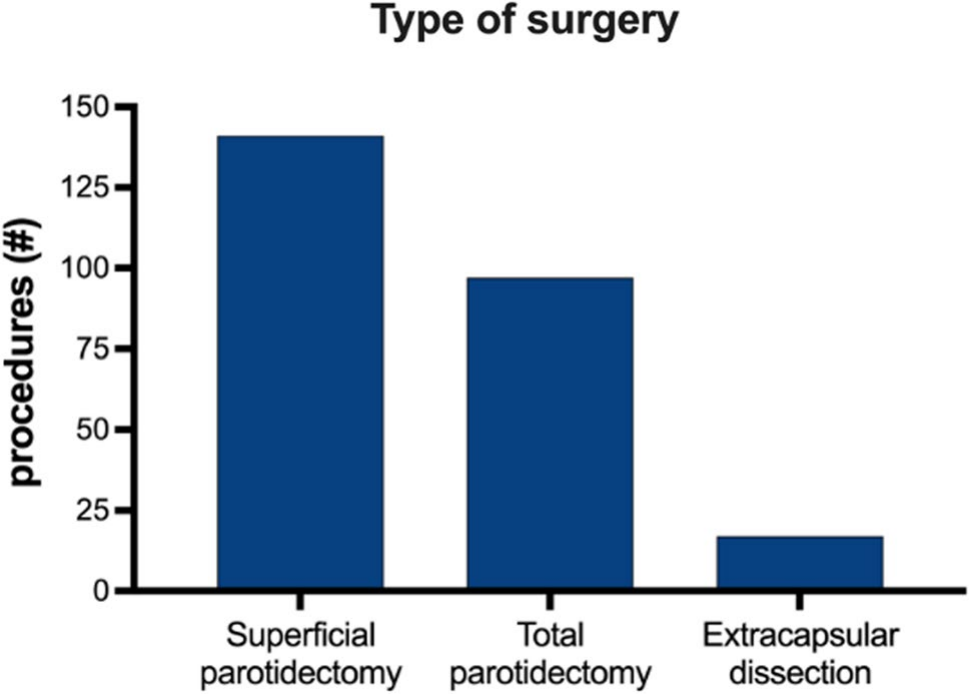
Percentage of different surgery performed

In our study, we preform the modified facelift and Blair skin incision. After the incision, 17 patients (6.7%) underwent an extracapsular dissection (ECD), 141 patients (55.3%) were treated with a superficial parotidectomy (SP), and 97 patients (38%) underwent a total parotidectomy (TP) (Fig. 1). Intentional (partial) facial nerve resection was performed in 7 cases (2.7%) in which tumor had involved the facial nerve with no possibility to isolate it from the mass.

### Complications

**Fig. 2:**
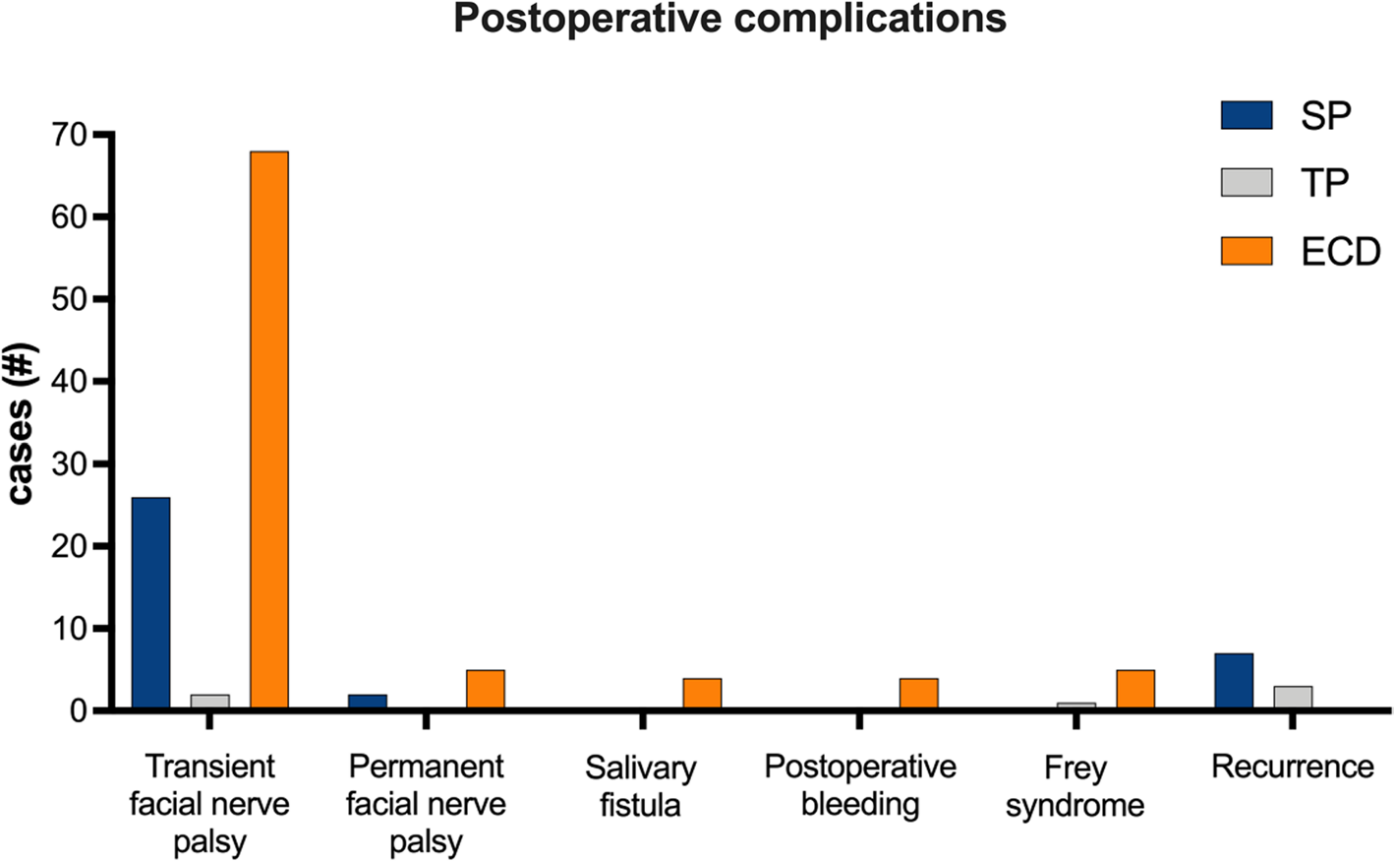
Post-operative complications

96 patients out of 255 (37.6%) had a transient facial nerve palsy (26 after a SP, 2 after ECD and 68 after TP) evaluated by the Original House-Brackmann classification and 7 patients (2.7%) reported a permanent facial nerve palsy (2 patients after SP and 5 after TP); 4 (1.6%) patients developed a salivary fistula; 4 (1.6%) a post-operative bleeding while 6 patients (2.3%) showed Frey Syndrome. Post-operative complications of ECD, SP and TP are described in Table 2 and Fig. 2.

**Table 2:** Post-operative complications (ECD: extracapsular dissection; SP: superficial parotidectomy; TP: total parotidectomy)

Post-operative complications	ECD	SP	TP
Transient facial nerve palsy	2 (0.8%)	26 (10.2%)	68 (26.7%)
Permanente facial nerve palsy	0	2 (0.8%)	5 (2%)
Frey syndrome	0	2 (0.8%)	4 (1.6%)
Post-operative bleeding	0	0	4 (1.6%)
Salivary fistula	0	0	4 (1.6%)

Although all histological results after the first operation were with margins R0, except two ECD patients that were R1, the observed rates of recurrence were significantly more frequent after ECD rather than when performing SP (70% vs 30% [*P* < 0.001]). No recurrences were noticed in case of TP. This result supports the fact that a more radical surgery may significantly reduce the recurrence rate, since in the TP more than in the SP and even more than in ECD a greater amount of glandular tissue from which a recurrence of the disease could have originated is removed. Transient facial nerve palsy, permanent facial nerve injury and Frey Syndrome were significantly more frequent after SP than after ECD (27% vs 2%; 28,5% vs 0%; 16.7% vs 0%, respectively [*P* < 0.001]). No salivary fistula or post-operative bleeding were observed after ECD or SP. All the complications which have been analyzed were more frequent after a TP. As concerns the nerve function recovery, all patients with partial nerve dysfunction recovered fully and it took an average time of 12 months for a full functional healing of the facial nerve.

To avoid Frey Syndrome or gustatory sweating, perioperative measures were taken such as closure of the superficial muscular aponeurotic system (SMAS), pedicle or free flap surgery, fat grafts and a sternocleidomastoid muscle flap.

The average post-surgical follow-up time was 20 months. As previously mentioned, recurrence was observed in 10 patients (3.4%), for 6 of them at 5-year follow-up, for 3 at 12-month follow-up and for 1 of them at 24-month follow-up. The other patients remained asymptomatic for all over the follow-up period. In case of recurrence, the patients underwent a more radical surgery choice: for example, in case of ECD, they underwent SP, while in case of SP, they experienced TP. In case of a third recurrence, the patient would have been referred to a radiotherapy treatment to complete the therapeutic procedure.

## Discussion

Pleomorphic adenomas are benign tumors generally seen in the superficial lobe of the parotid gland.

Most of the cases occur in individuals between 30 and 60 years old, with no gender predilection (at least for what concerned) in our population study.

Histologically, they can be easily recognized, consisting of epithelial and mesenchymal cells, but various patterns may be present in each mass. Pleomorphic adenoma is characterized by the presence of microscopic extension protruding beyond pseudocapsule or by capsular penetration [10]. Clinically, it presents itself as a slow-growing, painless tumor, which becomes malignant in 2–4% of cases [2]. The slow growth rate is probably the reason why pleomorphic adenomas are mostly asymptomatic, so that they are often discovered as incidental findings on radiographic imaging: in 2% of CT scans and in 20% of MRIs [11].

The symptomatology depends on the size, localization, and extension of the neoplasm. The most common signs at the time of diagnosis are represented by the presence of a mass in the parotid gland region and by pain with or without a preauricular mass. Less commonly, pleomorphic adenomas can lead to facial nerve involvement with loss of its function or the erosion of nearby structures [12].

The diagnosis of pleomorphic adenoma is both clinical and radiological. CT and MRI demonstrate a well-circumscribed mass occupying the parotid gland region or, less commonly, a mass growing out parotid gland region and involving the nearby structures with a variable density. MRI images help to outline the separation between the pleomorphic adenoma and the other surrounding tissue and should be performed when facial nerve involvement by the mass is suspected. MRI is currently used as the first-choice imaging tool considering its superior resolution that offers significant advantages for planning surgical treatment and for counseling patients about the most appropriate surgical treatment, its possible complications and post-operative care [11].

The choice of the surgical approach used to manage pleomorphic adenoma always depends on the size of the tumor and on its location, on the presence of complications, on the length of surgical time and on the surgeon's personal experience.

Approaches to pleomorphic adenomas can be divided into an extracapsular dissection (ECD), a partial superficial parotidectomy (PSP), a complete superficial parotidectomy (CSP), a partial superficial/deep lobe parotidectomy (PSDP) and a total parotidectomy (TP) technique [13].

Simple enucleation of a pleomorphic adenoma is associated with a high rate of recurrence which is reduced with superficial parotidectomy and further down with total parotidectomy. For this reason, superficial parotidectomy or total parotidectomy are generally preferred over enucleation. In fact, the SP is the surgical treatment that, based on our clinical experience, we recommend to perform in case of suspected pleomorphic adenoma.

Pleomorphic adenoma of the parotid gland appears as a single mass at initial presentation. In contrast, recurrent masses present as multinodular lesions. Recurrent tumors usually appear near the surgical scar from previous resections. There is no variety in recurrence rate according to gender or age. To avoid recurrence, as universally supported in literature, an appropriate surgical technique should be selected. Indeed, the most important reason for recurrence is enucleation with tumor rupture, incomplete resection during surgery or the existence of satellite lesions surrounding the capsule [2, 14]. A strategy to reduce the rate of recurrence could be to re-evaluate surgical margins using intraoperative frozen sections or remove the tumor with wide resection margins (a SP is safer than an ECD). In addition, the optimal regimen should involve total parotidectomy rather than enucleation or superficial parotidectomy, with the sacrifice of all parotid gland [15]. The recurrence rate of benign tumor is very low (3.4%), and the first recurrence is estimated in literature to occur on average 59–75 months after primary surgery and the secondary recurrence on average 25 months after surgery for the first relapse [2].

Regular ultrasound monitoring is a necessary post-operative practice for patients who received local resection or had risk factors such as multinodular masses.

Even though a complete removal of the parotid gland decreases the risk of recurrence, the major disadvantage concerns the potential complication for injuries of the facial nerve. Scarring of the surrounding tissues determines anatomical changes that make it harder to distinguish between normal tissue and tumor with an increased risk of facial nerve damage. Facial nerve integrity monitoring can reduce morbidity in case of recurrence or in case of TP.

For repeated resection of recurrent tumors, facial paralysis is present in about half of patients, with most recovering facial expressions within 1 year or less while some of facial paralysis were found to be permanent [16].

Radiotherapy could be an option for post-operative treatment of recurrent pleomorphic adenoma, especially when multinodular masses were present, facial nerves must be sacrificed, multiple recurrences have occurred, or complete excision is practically difficult. However, some authors have suggested that radiotherapy should be kept as the last resort management of malignant transformation [17].

According to the European literature, the analysis of our experience had similar data for mean age and sex distribution.

Regarding ECD and SP, the most frequently used approach, the current literature is conflicting in terms of disease recurrence and complications. Correlation of the surgical procedure with post-operative complications demonstrated transient and permanent facial nerve palsy in 2% and 0%, respectively, for ECD, 27% and 28.5% for SP and 71% and 71.5% for TP. These findings suggest that permanent nerve damage was more often associated with TP. Pharmaceutical and surgical treatment options for iatrogenic facial nerve palsies are limited and no uniform approach is currently available [16].

The incidence of Frey Syndrome or gustatory sweating was determined to be about 1.2%. It is suspected that the limited prevalence of gustatory sweating was at least partially due to faulty recognition of the clinical anamnestic features of Frey Syndrome. The time-to-onset of Frey Syndrome reported in the current literature varies from a few months to more than 4 years, depending on the diagnostic procedure [18]. Complication-free patients with a benign tumor did frequently not return to the surgeon after short-term follow-up, potentially explaining the lower prevalence rates when compared to the literature. The use of SMAS closure in preventing Frey Syndrome is currently controversial. It also enhances the esthetic outcome. Transposition of a sternocleidomastoid muscle flap reduces the risk of Frey Syndrome, and this also contributes to the esthetic outcome [19]. Dermal or free fat grafts play a role in preventing Frey Syndrome too [20]. Botulinum toxin A injection is used for the treatment of gustatory sweating [21].

In conclusion, clinicopathological knowledge of parotid tumors is essential for diagnostic assessment, treatment planning and patient counseling.

The clinical and statistical data did not show a statistically significant association between SP and the occurrence of at least one of the complications examined. For this reason, SP may be considered the treatment of choice for pleomorphic adenomas located in the superficial or deep portion of the parotid gland. SP, partial or complete, is recommended for tumors larger than 3.5 cm in diameter, when the lesion is in the deep or superficial lobe of the parotid gland or in case of tumor recurrence. The advantage of ECD, which is preferred when lesions are smaller than 3.5 cm, includes the removal of the mass with adequate margins of healthy parotid tissue and a reduction in the side effects after surgery preserving the parotid function even though it is associated to a higher risk of recurrence in our experience [22]. Total parotidectomy is the appropriate treatment of choice in most recurrences and in cases of large adenomas involving both deep and superficial parotid gland’s lobes.

A prolonged follow-up period is always recommended.

## Conclusions

On the basis of our experience, we suggest to always perform an accurate preoperative assessment of the patient to select the most suitable treatment to be radical in the removal of the tumor while minimizing the risk of complications and the rate of relapses. We highly recommend an accurate clinical evaluation, a complete anamnestic collection, and a complete otolaryngology physical examination. Subsequently, we recommend performing an ultrasound of the salivary glands involved and, if possible, to perform a CEUS. If the tests are suggestive for a diagnosis of pleomorphic adenoma, we recommend executing FNAC to confirm or exclude the diagnosis of pleomorphic adenoma, and, subsequently, an MRI to study the dimensions and the relationships of the tumor preoperatively.

These considerations are functional to the surgeon to choose the best therapeutic and personalized strategy for the patient, with the aim of being radical in the removal of the tumor with the lowest risk of intra and post-operative complications. If, despite a careful preoperative planning and an optimal choice and surgical technique, the pleomorphic adenoma relapses, we recommend performing a surgical radicalization (preferably using TP). At the third recurrence, in addition to a surgical revision of the parotid region, we suggest directing the patient towards radiotherapy treatment.

These indications, although already known in the literature, if correctly performed allow optimizing the surgical result as shown in our experience.
